# Antibacterial Activity of Defensin PaDef from Avocado Fruit (*Persea americana* var. *drymifolia*) Expressed in Endothelial Cells against *Escherichia coli* and *Staphylococcus aureus*

**DOI:** 10.1155/2013/986273

**Published:** 2013-11-12

**Authors:** Jaquelina Julia Guzmán-Rodríguez, Rodolfo López-Gómez, Luis M. Suárez-Rodríguez, Rafael Salgado-Garciglia, Luis C. Rodríguez-Zapata, Alejandra Ochoa-Zarzosa, Joel E. López-Meza

**Affiliations:** ^1^Centro Multidisciplinario de Estudios en Biotecnología, Facultad de Medicina Veterinaria y Zootecnia, Universidad Michoacana de San Nicolás de Hidalgo, Km 9.5 Carretera Morelia-Zinapécuaro, Posta Veterinaria, 58893 Morelia, MICH, Mexico; ^2^Instituto de Investigaciones Químico Biológicas, Universidad Michoacana de San Nicolás de Hidalgo (UMSNH), Ciudad Universitaria, Edificio B1, 58030 Morelia, MICH, Mexico; ^3^Unidad de Biotecnología, Centro de Investigación Científica de Yucatán, A.C. Calle 43 No. 130 Col. Chuburná de Hidalgo, 97200 Mérida, YUC, Mexico

## Abstract

Antimicrobial therapy is a useful tool to control infectious diseases in general and rising antibiotic resistant microorganisms in particular. Alternative strategies are desirable, and antimicrobial peptides (AMP) represent attractive control agents. Mexican avocado (*Persea americana* var. *drymifolia*) is used in traditional medicine; however, the AMP production has not been reported in this plant. We obtained a cDNA library from avocado fruit and clone PaDef was identified, which has a cDNA (249 bp) encoding a protein (78 aa) homologous with plant defensins (>80%). We expressed the defensin *PaDef* cDNA (pBME3) in the bovine endothelial cell line BVE-E6E7. Polyclonal and clonal populations were obtained and their activity was evaluated against *Escherichia coli*, *Staphylococcus aureus*, and *Candida albicans*. *E. coli* viability was inhibited with 100 **μ**g/mL of total protein from clones (>55%). Also, *S. aureus* viability was inhibited from 50 **μ**g/mL total protein (27–38%) but was more evident at 100 **μ**g/mL (52–65%). This inhibition was higher than the effect showed by polyclonal population (*~*23%). Finally, we did not detect activity against *C. albicans*. These results are the first report that shows antimicrobial activity of a defensin produced by avocado and suggest that this AMP could be used in the control of pathogens.

## 1. Introduction

The excessive and inappropriate use of conventional antibiotics in the clinical treatment of human and animal infections has increased pathogen resistance against these compounds, turning them into less effective agents. As a consequence, there has been an increase in the generation of multidrug-resistant pathogens, primarily bacteria and fungi that resist the effects of most antibiotics [1, 2]. Thus, alternative methods for controlling pathogens are required. In this sense, the plants are an attractive alternative because they exhibit a huge variety of compounds with antimicrobial activity.

The antimicrobial metabolites from plant origin derive mainly from secondary metabolism. These compounds include diverse groups such as saponins, phenolic compounds, cyclic hydroxamic acids, cyanogenic glycosides, isoflavonoids, sesquiterpenes, and sulfur-containing indole derivatives [3]. Avocado (*Persea americana *Mill.) is an important worldwide agricultural crop native of Mexico and Central America, which contains diverse metabolites with antimicrobial activity. For example, the 1,2,4-trihydroxy-n-heptadeca-16-en isolated from avocado fruit and seeds showed antibacterial activity [4]. Also, the presence of antifungal dienes from idioblast cells and exocarp and mesocarp of unripe fruits has been described [5–7]. In addition, metabolites with antiviral activity from avocado leaves have been reported [8, 9]. More recently, Sánchez-Pérez et al. [10] showed that crude root extracts from *P. americana* var. *drymifolia* inhibit the mycelial growth of *Phytophthora cinnamomi*, this activity was attributable to stigmastan-3, 5-diene. The great compound diversity with antimicrobial properties in avocado tissues is evident; despite this, the production of antimicrobial peptides (AMPs) by* P. americana* has not been reported.

AMPs are produced by a wide variety of organisms as part of their first line of defense (eukaryotes) or as a competition strategy for nutrients and space (prokaryotes) and have a broad and nonspecific activity that makes them strong candidates for a variety of pharmacological applications [11].

Plants produce AMPs as part of their defense mechanisms; these can be expressed constitutively or induced in response to pathogen attack [12, 13]. Plant AMPs have a molecular weight in the range of 2 to 10 kDa, are basic, and contain 4, 6, 8, or 12 cysteines that form disulfide bonds [13, 14]. Plant AMPs are classified in 10 families; one of the most important are the defensins, which are basic AMPs with an approximate molecular weight of 5 kDa (45 to 54 amino acids); they typically have eight cysteines and exhibit mainly antifungal activity [12, 13].

In previous works, we have reported that the expression of AMPs in bovine endothelial cells is a useful tool to explore antibacterial, fungicidal, and cytotoxic activities against a broad range of mammal pathogens and human tumor cell lines [15, 16]. In this study, we isolated the cDNA of defensin *PaDef *from avocado fruit, which was expressed as a fusion protein in the endothelial cell line BVE-E6E7. We show that the conditioned media (CM) from these cells have antimicrobial activity against *Escherichia coli* and *Staphylococcus aureus*, important pathogens from animals and humans.

## 2. Materials and Methods

### 2.1. Biological Material and Bacterial Strains

Avocado fruits of *Persea americana* var. *drymifolia* “native Mexican” were collected in the Germplasm Bank of the Instituto Nacional de Investigaciones Forestales y Agropecuarias (INIFAP; Uruapan, Michoacán, Mexico). Fruits were cut and immediately frozen in liquid nitrogen. Seeds were separated from the rest of the fruit tissue.

As representative of gram-negative bacteria, we used the enteropathogenic *Escherichia coli* strain 0111 from Instituto Nacional de Referencia Epidemiológica (SSA, Mexico) that was kindly donated by M.S. Vázquez-Garcidueñas (UMSNH, Mexico). The American Type Culture Collection (ATCC) *S. aureus* subsp. *aureus *27543 strain isolated from a case of clinical mastitis was used as representative of gram-positive bacteria. Inoculum was prepared from bacteria that were grown at 37°C overnight in Luria-Bertani broth (LB, Bioxon, Mexico). Additionally, the ATCC *Candida albicans *10231 strain was used. In this case, the inoculum was prepared from fungus that was grown at 21°C for 48 h in YPD broth (2% dextrose, 2% Bacto Peptone, 1% yeast extract; Difco).

### 2.2. Cell Cultures

The bovine endothelial cell line BVE-E6E7 immortalized with the human papillomavirus type 16 E6E7 oncogene was used to express defensin *PaDef* cDNA from *P. americana *var. *drymifolia* [17]. Cells were routinely cultured in Dulbecco's modified Eagle's medium (DMEM, Sigma), supplemented with 10% fetal calf serum (FCS, Equitech-Bio), 100 U/mL penicillin and streptomycin (Gibco) and grown in an atmosphere of 5% CO_2_ at 37°C.

### 2.3. cDNA Library Construction and Sequencing by Sanger Method

Total RNA from frozen pulp tissue was extracted using R. López-Gómez and M. A. Gómez-Lim. [18] protocol with some modifications. All cDNA complementary libraries were built from 1 *μ*g of total RNA using SMART cDNA library construction Kit (Clontech). The obtained cDNA sequences were cloned into TripIEx2 vector. Excision experiments were made using *E. coli* BM25.8 cells to obtain the plasmid pTriplEx2. Sequencing reactions were performed using ABI PRISM BigDye Terminators v3.0 kit (Applied Biosystems) by 5′ end of plasmids extracted from random clones. The sequences obtained were filtered by quality using PHRED [19]; vector masked and trimming of poly A/T were performed using LUCY2 software [20]. Identification of defensin *PaDef* was reported elsewhere [21].

### 2.4. Bioinformatic Analysis of the Defensin PaDef from Avocado

The nucleotide sequence of defensin *PaDef *cDNA and the deduced amino acid sequence were analyzed using the Blast tool in the NCBI (http://blast.ncbi.nlm.nih.gov/). The peptide structure was evaluated for the presence of a signal peptide sequence with SignalP (http://www.cbs.dtu.dk/services/SignalP/), and the possible disulfide bridge pattern was determined using the DISULFIND predictor (http://disulfind.dsi.unifi.it/).

The deduced amino acid sequence encoding for the plant defensin peptide was aligned against sequences of defensin holotype type 1 (*Raphanus sativus *Rs-AFP1, GenBank: AAA69541.1) and defensin type 2 (*Nicotiana alata* NaD1, GenBank: AAN70999.1) using ClustalX [22]. Homology models for defensin PaDef peptide were created using the Protein Model Portal (http://www.proteinmodelportal.org). The crystal structure of Rs-AFP1 (Protein Data Bank: 1AYJ) from radish was used as template. The models obtained were refined and analyzed with YASARA tools (http://www.yasara.org).

### 2.5. Construction of Expression Vector and Transfection of BVE-E6E7 Cells

For transfection assays the defensin *PaDef* cDNA was cloned into the mammalian expression vector pTracer-EF/V5-His-A (Invitrogen), and the construction was denominated pBME3. To obtain this construction, the defensin *PaDef* cDNA was amplified by PCR with specific primers that were modified with restriction sites to facilitate the manipulation. The primers (Invitrogen) were: forward, 5′-TATT**ACTAGT**ATGGCGCTGGTCAAGAAG-3; reverse, 5′-ATAA**GAATTC**GCAAGGCTTGAGACACA-3′; in bold are indicated the restriction sites* Spe*I and *Eco*RI, respectively. Amplifications were performed under the following conditions: 5 min of initial denaturation at 94°C followed by 30 cycles of amplification at 94°C for 30 s, 52°C for 30 s, and 72°C for 1 min. The amplification product (249 bp) was subcloned into the *Spe*I and *Eco*RI sites of pTracer-EF/V5-His-A vector. This vector contains the resistance gene for zeocin and the reporter gene for the green fluorescent protein (GFP), which allows the selection of transfected cells by antibiotic resistance and fluorescence microscopy, respectively. The correct fusion was confirmed by sequencing. BVE-E6E7 cells were stably transfected with the construction pBME3 or pTracer EF/V5-His-A vector (negative control) by lipofection using Lipofectamine 2000 (Invitrogen) according to manufacturer's instructions. Polyclonal populations of transfected cells were selected in Opti-mem medium (Gibco) containing 500 *μ*g/mL zeocin (Invitrogen) for 2 weeks and by expression of GFP. Cells were maintained with 200 *μ*g/mL zeocin after selection. Next, 9 clonal populations were obtained by limiting dilution and were analyzed through this study. Expression of defensin *PaDef* by transfected BVE-E6E7 cells was analyzed by RT-PCR as described previously [23, 24].

### 2.6. Transfected Cells Conditioned Media

To obtain the conditioned media, polyclonal and clonal populations of BVE-E6E7 cells transfected with pBME3 (BVE3) or pTracer-EF/V5-His-A (BVpT) that corresponds to empty vector were grown at confluence in p100 Petri dishes (Costar). Culture medium was replaced with 10 mL of Optimem medium (Gibco) without serum and antibiotics, and the cells were cultured for 24 h. Conditioned media were clarified by centrifugation (10 min, 1200 ×g). The concentration of total protein was determined by the Bradford method.

### 2.7. Viability Assays of *Staphylococcus aureus*, *Escherichia coli*, and *Candida albicans* Strains

5 × 10^4^ CFU of *S. aureus* and 3 × 10^4^ CFU of *E. coli* were incubated with different concentrations ranging from 10 to 100 *μ*g/mL of total protein of CM from polyclonal and clonal populations during 4 h at 37°C in a 96-well flat-bottom plate. Then, 10 *μ*L of 5 mg/mL of 3-(4,5-dimethyl-2-thiazolyl)-2,5-diphenyl-2H-tetrazolium bromide (MTT, Sigma) solution in PBS was added to each well and incubated for 4 h at 37°C. After that, 100 *μ*L of acid isopropanol (95% isopropanol and 5% of 1 N HCl) was added to dissolve formazan crystals. Optical density was measured with a microplate spectrophotometer (DAS) at 595 nm [25]. Wells containing Optimem medium were used as background controls. Cells treated with gentamicin (40 *μ*g/mL, Sigma) were used as negative control of viability. All assays were run in triplicates. In the case of *C. albicans*, 1 × 10^5^ cells were incubated with the same concentrations of total protein for 24 h at 37°C and then evaluated as described for bacteria. Cells treated with amphotericin (250 ng/mL, Sigma) were used as negative control of viability.

### 2.8. Flow Cytometry Analysis

BVE3-C1 cells were plated at confluence in 24-well tissue culture plates. Then, monolayers were detached with trypsin-EDTA (Sigma) and were transferred to 1.5 mL microtubes. Cells were centrifuged at 2500 rpm and washed with PBS, and the pellet was fixed with 4% paraformaldehyde in PBS for 10 min at 4°C. Then, cells were blocked with 5% normal goat serum (Sigma) for 30 min on ice. Cells were permeabilized with 0.1% Triton X-100 for 10 min at 4°C and were incubated with primary antibody anti-V5 epitope (1 : 500, Invitrogen) overnight at 4°C and finally with the TRITC conjugated-secondary antibody against mouse IgG (1 : 50, Molecular Probes) for 45 min on ice. Cells were washed three times with PBS-Triton and analyzed in an Accuri C6 flow cytometer (Accuri Cytometers) using CFlow software. BVpT were used as control, or BVE3-C1 cells were incubated only with secondary antibody.

### 2.9. Data Analysis

Data were compared by analysis of variance and Student's *t*-test. The results are reported as mean ± the standard errors (SE). *P* values of <0.05 were considered significant.

## 3. Results

### 3.1. Characteristics of Defensin PaDef from Avocado Fruit

**Figure 1:**
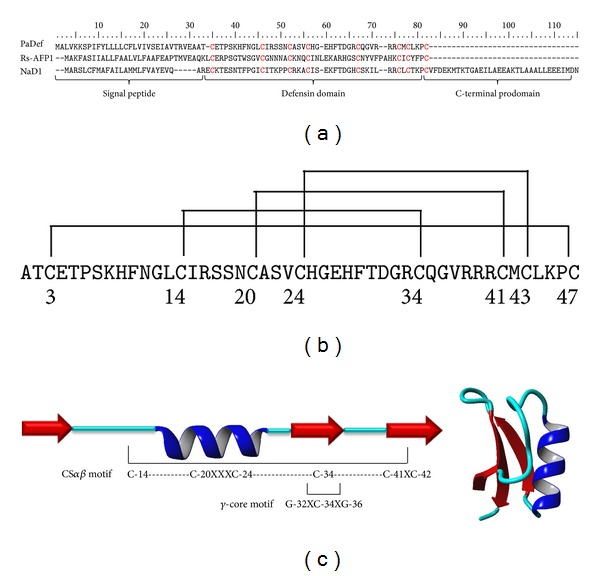
Characteristics of defensin PaDef from *Persea americana* var. *drymifolia*. (a) Alignment of the defensin PaDef with defensin holotype type 1 (*R. sativus* Rs-AFP1) and defensin holotype type 2 (*N. alata* NaD1). In red, the 8 conserved cysteines are indicated. (b) Prediction of disulfide bridges of defensin PaDef. The pattern was determined using the DISULFIND predictor. (c) Structural organization of defensin PaDef peptide. Left: secondary structure showing the characteristic CS*αβ* motif of defensins. Red arrows indicate the *β*-sheets. Right: homology model of defensin PaDef peptide. Model was created using Rs-AFP1 (Protein Data Bank: 1AYJ) as template.

In order to analyze the genes expressed in avocado fruit, R. López-Gómez et al. [21] prepared an EST library from the pulp of *P. americana* var. *drymifolia* fruit. From this library, one clone was identified and further characterization showed that it contains a cDNA with homology to plant AMPs, which was designated defensin PaDef (Accession GenBank KC007441). Sequence analysis of this clone revealed that it has a cDNA of 249 bp and one putative open reading frame (ORF) with a protein coding capacity of 78 amino acids (5.2 kDa). A bioinformatic analysis showed that the amino acid sequence of the ORF has homology (>80%) with plant defensins. This putative protein contains a characteristic signal peptide of 31 aa, which when removed produces a mature peptide of 47 aa. Also, an alignment with defensin holotype type 1 (*R. sativus* Rs-AFP1) and type 2 (*N. alata* NaD1) allowed us to classify it as type 1 defensin (Figure 1(a)). A comparative study with plant defensins showed that this ORF contains the conserved 8 cysteines, which could form the 4 disulfide bridges characteristic of these AMPs (Figure 1(b)). Also, the structure contains the CS*αβ* and *γ*-core motifs present in these defensins (Figure 1(c)). From these analysis it was established that the ORF identified in the clone PaDef encodes a defensin from avocado.

### 3.2. Expression of Avocado Defensin PaDef in BVE-E6E7 Cells

**Figure 2:**
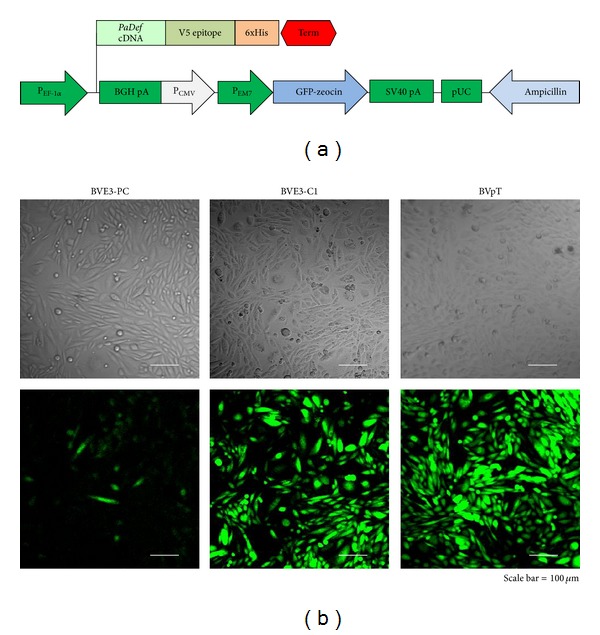
Transfection of BVE-E6E7 endothelial cells with pBME3 construction. (a) The pBME3 construction contains the EF1-*α* promoter, defensin *PaDef *cDNA, CMV promoter, EM7 promoter, GFP-zeocin resistance gene, and SV40 polyadenylation sequence. (b) Polyclonal, population (BVE3-PC) and representative cloned population (BVE3-C1) of BVE-E6E7 cells transfected with pBME3 visualized under light microscopy (upper) and fluorescence (below). Also, the control cells are showed (BVpT). Scale bar: 100 *μ*m.

**Figure 3:**
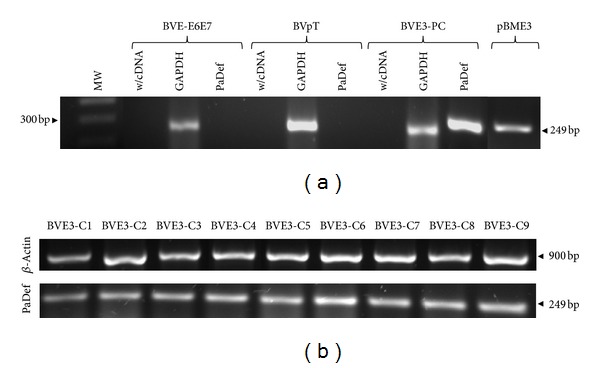
mRNA expression of defensin PaDef of avocado in BVE-E6E7 endothelial cells. (a) RT-PCR analysis that shows the amplification of defensin *PaDef* in polyclonal population of BVE-E6E7 cells transfected (BVE-PC). The lack of defensin *PaDef *amplification is shown in nontransfected BVE-E6E7 cells (BVE-E6E7) or only transfected with the vector (BVpT). Also, the positive control is included (pBME3). The 1 kb molecular weight marker (Invitrogen) was also included (MW). (b) RT-PCR analysis that shows the amplification of defensin *PaDef *in different endothelial cell clones.

**Figure 4:**
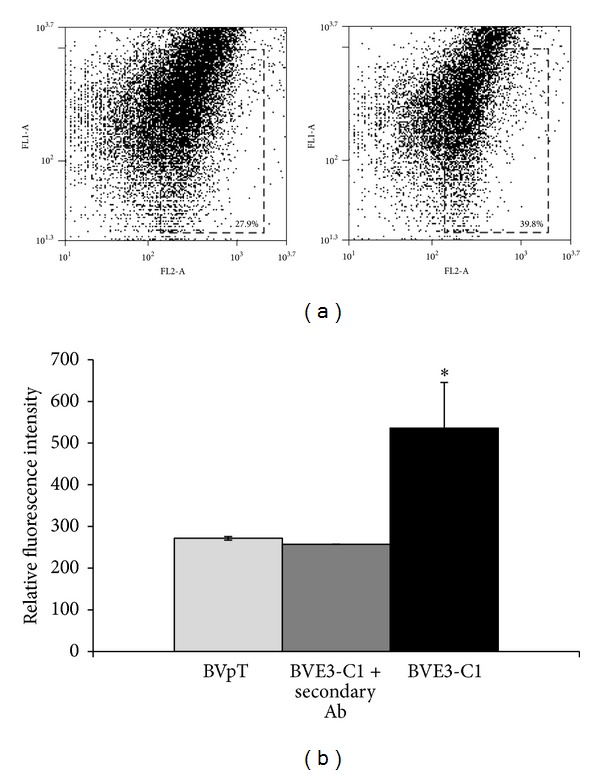
Defensin PaDef expression in BVE-E6E7 endothelial cells analyzed by flow cytometry. (a) Left: scatter plot of clonal population of BVE-E6E7 cells transfected with BVpT (control cells). Right: scatter plot of clonal population of BVE-E6E7 cells that express the defensin PaDef (BVE3-C1). The values inside rectangle indicate the percentage of cells that displayed fluorescence intensity higher than 2000 relative units. (b) Relative fluorescence intensity of BVE-E6E7 control cells (BVpT) and BVE-E6E7 cells that express the defensin PaDef (BVE3-C1). Fluorescence intensity was estimated from 5500 events. A control of BVE-E6E7 cells that express the defensin PaDef (BVE3-C1) only treated with the secondary antibody was included (BVE3-C1 + secondary Ab). Values of relative fluorescence intensity were obtained subtracting the fluorescence intensity corresponding to GFP expression. *Significant changes (*P* < 0.05) compared to BVE-E6E7 control cells.

To express defensin *PaDef* from avocado in BVE-E6E7 cells, we used the pBME3 construction (Figure 2(a)). This construction was introduced into BVE-E6E7 cells by lipofection. The human elongation factor 1-*α* (EF1-*α*) promoter directs the expression of defensin PaDefcDNA in BVE-E6E7, while the human cytomegalovirus promoter (CMV) directs the expression of the selection marker (GFP-zeocin resistance gene fusion). Initially, we obtained a polyclonal population of BVE-E6E7 cells transfected with pBME3 construction (BVE3-PC) (Figure 2(b)). Further, with the aim to obtain homogeneous BVE-E6E7 cells transfected with pBME3, a total of 9 clones were selected from the polyclonal population using limiting dilution (BVE3-C1 to C9 clones) (Figure 2(b)). *PaDef* mRNA expression was demonstrated by RT-PCR analysis. An amplicon of ~250 bp corresponding to defensin *PaDef* was observed in polyclonal population of BVE-E6E7 cells transfected with pBME3 and the clones selected (Figures 3(a) and 3(b)), this amplicon was also obtained in the positive control (Figure 3(a), pBME3). Specific amplification was confirmed by sequencing the PCR products and by the absence of amplification in the negative control (Figure 3(a), BVpT). Also, defensin *PaDef* mRNA expression analysis of the clones showed that these have similar expression levels (Figure 3(b)). In addition, the expression of PaDef was analyzed in clone BVE3-C1 by flow cytometry using the antibody anti-V5 epitope (Figure 4). This clone showed higher relative fluorescence intensity compared to control cells, indicating that the protein of defensin PaDef is expressed in these cells (Figure 4(b)).

Further, CM from polyclonal and clonal populations were obtained as described in materials and methods and were used to assess if it have any effect on viability of BVE-E6E7 cells, which was evaluated by trypan blue exclusion technique. Results showed that none of the treatments significantly decreased cell viability (data not shown).

### 3.3. Antimicrobial Activity of CM from BVE-E6E7 Cells That Express Defensin PaDef from Avocado

CM from polyclonal and clonal populations were used to evaluate their antibacterial activity against *S. aureus *ATCC 27543 and *E. coli* 0111 strains. The defensin *PaDef *cDNA used to transfect BVE-E6E7 cells encodes for a signal peptide of 31 aa located at the N-terminal of the mature peptide, suggesting that defensin PaDef might be secreted to culture medium. Bacteria cells were incubated with several concentrations of total protein from CM, and their viability was estimated by MTT assay. According to previous works, we evaluated diverse concentrations of total protein of CM, ranging from 10 to 100 *μ*g/mL. Results showed that *E. coli *viability was inhibited when bacteria were challenged with 100 *μ*g/mL of total protein of CM from the different clones (>55%). This effect was similar for all the clones evaluated (Table 1). These values were higher than the effect showed by CM from polyclonal population (~20%). In the rest of the conditions tested we did not detect significant differences.

Regarding the effects on *S. aureus* viability, we observed an inhibition of viability from 50 *μ*g/mL total protein (27–38%) when bacteria were treated with CM from clones (Table 1). This effect was more evident at 100 *μ*g/mL of total protein; in this case the bacterial viability was inhibited at 52–65%. Similarly the effect showed for* E. coli*,CM from polyclonal population only inhibited the 23% of *S. aureus* viability at 100 *μ*g/mL of total protein. Similar results of antibacterial activity were obtained when we compared the effect of CM from BVE-E6E7 cells expressing defensin PaDef against CM from BVE-E6E7 nontransfected (Table 1) or CM from BVE-E6E7 transfected with BVpT (data not shown). On the other hand, we did not detect antifungal activity against *C. albicans*. These results have shown that CM from BVE-E6E7 cells expressing defensin PaDef from avocado have antibacterial activity against human and animal pathogens.

## 4. Discussion

In this study we assessed the antimicrobial activity of defensin PaDef from *P. americana* var. *drymifolia *fruit expressed in bovine endothelial cells. In previous works, we have shown that the bovine endothelial cell line BVE-E6E7 is a valuable expression system to evaluate the antimicrobial activities of plant AMPs [15, 23]. Avocado is a worldwide important crop; in addition to its use as food, it is utilized in traditional medicine due to its curative properties [26]. Several avocado metabolites (essentially from secondary metabolism) are known to have antibacterial, antifungal, and insecticidal activity; however, very little is known about AMPs from this plant [4, 26–28].

Plant defensins have been isolated from many species and represent an alternative to agricultural biotechnology and therapeutic drug design [13]. In this work, we obtained the avocado *PaDef *cDNA from fruit. Alignment analysis of this cDNA in the NCBI database revealed a high homology (>80%) with plant AMP. Analysis of deduced amino acid sequences shows that the peptide encoding a protein (78 aa) shared the common structure of defensins, including a signal peptide of 31 aa (Figure 1). Most plant defensins are expressed as a prepeptide with N-terminal region containing a signal peptide for extracellular secretion [29]. Alignment analysis of the mature region (47 aa) showed that the defensin PaDef peptide contains 8 cysteines, which could form 4 disulfide bridges (Figure 1) and shares high similarity at deduced amino acid level with type I defensins [13]. The three-dimensional structure of plant defensins presents a CS*αβ* motif consisting of a triple stranded, anti-parallel beta-sheet, and one alpha-helix following a *β* *αβ* *β* pattern, which is stabilized by disulfide bridges [29]. This motif was also identified in defensin PaDef from avocado (Figure 1(c)). Also, defensin PaDef contains the *γ*-core motif, which is important to structure stabilization and has been associated with antifungal activity [30]. To our knowledge, this is the first report that shows the identification of a defensin in avocado fruit.

The defensin *PaDef *cDNA used to transfect BVE-E6E7 cells encodes for a signal peptide of 31 aa located at the N-terminal of the mature peptide suggesting that defensin PaDef might be secreted by cells to culture medium. The expression of this cDNA in polyclonal and clonal populations was demonstrated by mRNA analysis and corroborated by sequencing (Figure 3). Also, defensin PaDef protein expression was corroborated by flow cytometry (Figure 4). We tested the antibacterial activity of the CM from all clones by the MTT assay (Table 1). In general, we did not detect significant differences in antibacterial activity between the clones, which is directly related to the fact that mRNA expression is also homogeneous in clones. In respect to the effect of CM against *E. coli*, we showed that the CM from clones had a clear inhibitory effect at 100 *μ*g/mL of total protein. On the other hand, the antibacterial activity against *S. aureus* was concentration-dependent. We attribute these differential effects to the differences in the structure of the membrane and cell walls of these organisms.

It has been established that the main activity of plant defensins is antifungal [12]. Interestingly, we did not detect antifungal activity against *C. albicans*. In agreement, Segura et al. [31] report that several defensins of spinach (So-D1, 2, 6, and 7) exhibit antibacterial activity but not fungicide activity, evaluated against *Fusarium culmorum* and *F. solani*. However, further studies are needed to evaluate a wider group of fungi, including avocado fungal pathogens in order to establish the antifungal activity of avocado defensin.

## 5. Conclusion

The present data is the first report that shows antimicrobial activity of a defensin produced by avocado fruit. Overall, the results of this study suggest that defensin PaDef from avocado is an AMP that could be used in the treatment of infectious diseases.

## Figures and Tables

**Table 1:** Antibacterial effect of CM from BVE-E6E7 cells that express defensin PaDef from *Persea americana *var. *drymifolia* ^a^.

	Inhibition (%)^b^							
Clones	*E. coli* ^ c^				*S. aureus* ^ c^			
	CM (total protein, *μ*g)				CM (total protein, *μ*g)			
	10	25	50	100	10	25	50	100
BVE3-PC	5.69	14.06	1.2	20.4	11.06	10.29	24.69	23.57
BVE3-C1	8.82	1.02	11.49	73.67*	4.75	4.33	38.87*	64.26*
BVE3-C2	10.37	9.74	10.51	69.64*	17.13	12.83	33.96*	63.73*
BVE3-C3	12.99	0.87	6.78	75.79*	11.64	14.35	30.86*	67.43*
BVE3-C4	23.56	15.44	14.91	65.37*	20.27	15.37	33.23*	54.59*
BVE3-C5	21.81	15.89	11.81	75.71*	13.77	6.14	30.25*	64.95*
BVE3-C6	20.26	16	7.73	78.08*	12.53	10.52	31.29*	63.86*
BVE3-C7	13.8	13.11	20.87	79.5*	11.97	3.87	31.07*	59.67*
BVE3-C8	22.15	13.4	9.56	70.88*	15.93	10.71	32.62*	58.41*
BVE3-C9	16.29	14.56	9.22	55.62*	1.47	14.45	27.02*	52.23*

^a^Bacteria were incubated during 4 h at 37°C with CM.

^b^Percentage of inhibition considering the effect of CM from BVE-E6E7 cells as 100% for each strain.

^c^Data represent the mean value ± SE of three independent experiments.

*Significant changes (*P* < 0.05) compared to CM from BVE-E6E7 cells.

## References

[1] Heuer OE, Hammerum AM, Collignon P, Wegener HC (2006). Human health hazard from antimicrobial-resistant enterococci in animals and food. *Clinical Infectious Diseases*.

[2] Field D, Quigley L, O’Connor PM (2010). Studies with bioengineered Nisin peptides highlight the broad-spectrum potency of Nisin V. *Microbial Biotechnology*.

[3] Papadopoulou K, Melton RE, Leggett M, Daniels MJ, Osbourn AE (1999). Compromised disease resistance in saponin-deficient plants. *Proceedings of the National Academy of Sciences of the United States of America*.

[4] Néeman I, Lifshitz A, Kashman Y (1970). New antibacterial agent isolated from the avocado pear. *Applied microbiology*.

[5] Prusky D, Keen NT, Sims JJ, Midland SL (1982). Possible involvement of an antifungal diene in the latency of *Colletotrichum gloeosporioides* on unripe avocado fruits. *Phytopathology*.

[6] Prusky D, Keen NT (1993). Involvement of preformed antifungal compounds in the resistance of subtropical fruits to fungal decay. *Plant Disease*.

[7] Domergue F, Helms GL, Prusky D, Browse J (2000). Antifungal compounds from idioblast cells isolated from avocado fruits. *Phytochemistry*.

[8] Wigg MD, Al-Jabri AA, Costa SS, Race E, Bodo B, Oxford US (1996). *In vitro* virucidal and virustatic anti HIV-1 effects of extracts from *Persea americana* Mill. (avocado) leaves. *Antiviral Chemistry and Chemotherapy*.

[9] de Almeida AP, Miranda MM, Simoni IC, Wigg MD, Lagrota MHC, Costa SS (1998). Flavonol monoglycosides isolated from the antiviral fractions of *Persea americana* (Lauraceae) leaf infusión. *Phytotherapy Research*.

[10] Sánchez-Pérez JDL, Jaimes-Lara MG, Salgado-Garciglia R, López-Meza JE (2009). Root extracts from Mexican avocado (*Persea americana* var. *drymifolia*) inhibit the mycelial growth of the oomycete *Phytophthora cinnamomi*. *European Journal of Plant Pathology*.

[11] López-Meza JE, Ochoa-Zarzosa A, Aguilar JA, Loeza-Lara PD, Komorowska MA, Olsztynska-Janus S (2011). Antimicrobial peptides: diversity and perspectives for their biomedical application, biomedical engineering, trends, research and technologies. *Biomedical Engineering, Trends, Research and Technologies*.

[12] Thomma BPHJ, Cammue BPA, Thevissen K (2002). Plant defensins. *Planta*.

[13] Lay FT, Anderson MA (2005). Defensins—components of the innate immune system in plants. *Current Protein and Peptide Science*.

[14] García-Olmedo F, Rodríguez P, Molina A (2001). Antibiotic activities of peptides, hydrogen peroxide and peroxynitrite in plant defence. *FEBS Letters*.

[15] Anaya-López JL, López-Meza JE, Baizabal-Aguirre VM, Cano-Camacho H, Ochoa-Zarzosa A (2006). Fungicidal and cytotoxic activity of a *Capsicum chinense* defensin expressed by endothelial cells. *Biotechnology Letters*.

[16] Loeza-Ángeles H, Sagrero-Cisneros E, Lara-Zárate L, Villagómez-Gómez E, López-Meza JE, Ochoa-Zarzosa A (2008). Thionin Thi2.1 from *Arabidopsis thaliana* expressed in endothelial cells shows antibacterial, antifungal and cytotoxic activity. *Biotechnology Letters*.

[17] Cajero-Juárez M, Avila B, Ochoa A (2002). Immortalization of bovine umbilical vein endothelial cells: amodel for the study of vascular endothelium. *European Journal of Cell Biology*.

[18] López-Gómez R, Gómez-Lim MA (1992). A method for extracting intact RNA from fruits rich in polysaccharides using ripe mango mesocarp. *HortScience*.

[19] Ewing B, Green P (1998). Base-calling of automated sequencer traces using phred. II. Error probabilities. *Genome Research*.

[20] Li S, Chou H-H (2004). Lucy2: an interactive DNA sequence quality trimming and vector removal tool. *Bioinformatics*.

[21] López-Gómez R, Ibarra LE, Suárez RLM First insights into the avocado fruit transcriptome.

[22] Larkin MA, Blackshields G, Brown NP (2007). Clustal W and Clustal X version 2.0. *Bioinformatics*.

[23] Ochoa-Zarzosa A, Loeza-Ángeles H, Sagrero-Cisneros E, Villagómez-Gómez E, Lara-Zárate L, López-Meza JE (2008). Antibacterial activity of thionin Thi2.1 from *Arabidopsis thaliana* expressed by bovine endothelial cells against *Staphylococcus aureus* isolates from bovine mastitis. *Veterinary Microbiology*.

[24] Alva-Murillo N, Ochoa-Zarzosa A, López-Meza JE (2012). Short chain fatty acids (propionic and hexanoic) decrease *Staphylococcus aureus* internalization into bovine mammary epithelial cells and modulate antimicrobial peptide expression. *Veterinary Microbiology*.

[25] Jahn B, Martin E, Stueben A, Bhakdi S (1995). Susceptibility testing of *Candida albicans* and *Aspergillus* species by a simple microtiter menadione-augmented 3-(4,5-dimethyl-2-thiazolyl)-2,5- diphenyl-2H-tetrazolium bromide assay. *Journal of Clinical Microbiology*.

[26] Yasir M, Das S, Kharya M (2010). The phytochemical and pharmacological profile of *Persea americana* Mill. *Pharmacognosy Reviews*.

[27] King JR, Knight RJ (1987). Occurrence and assay of estragole in the leaves of various avocado cultivars. *Journal of Agricultural and Food Chemistry*.

[28] Rodríguez-Saona C, Trumble JT (2000). Secretory avocado idioblast oil cells: evidence of their defensive role against a non-adapted insect herbivore. *Entomologia Experimentalis et Applicata*.

[29] Padovan L, Scocchi M, Tossi A (2010). Structural aspects of plant antimicrobial peptides. *Current Protein and Peptide Science*.

[30] Sagaram US, Pandurangi R, Kaur J, Smith TJ, Shah DM (2011). Structure-activity determinants in antifungal plant defensins msdef1 and mtdef4 with different modes of action against *Fusarium graminearum*. *PLoS ONE*.

[31] Segura A, Moreno M, Molina A, García-Olmedo F (1998). Novel defensin subfamily from spinach (*Spinacia oleracea*). *FEBS Letters*.

